# Relations between air pollution and vascular development in 5-year old children: a cross-sectional study in the Netherlands

**DOI:** 10.1186/s12940-019-0487-1

**Published:** 2019-05-16

**Authors:** Anna-Maria Ntarladima, Ilonca Vaartjes, Diederick E. Grobbee, Martin Dijst, Oliver Schmitz, Cuno Uiterwaal, Geertje Dalmeijer, Cornelis van der Ent, Gerard Hoek, Derek Karssenberg

**Affiliations:** 1 Julius Centre for Health Sciences and Primary Care, University Medical Centre Utrecht, Utrecht University, Utrecht, The Netherlands; 2 Department of Physical Geography, Faculty of Geosciences, Utrecht University, Utrecht, The Netherlands; 3 Global Geo Health Data Center, Utrecht University, Utrecht, The Netherlands; 4 Luxembourg Institute of Socio-Economic Research LISER, Esch-sur-Alzette Luxemburg, UK; 5 Netherlands Institute for Risk Assessment Sciences (IRAS), Utrecht University, Utrecht, The Netherlands; 6 Department of Pediatric Pulmonology, and Cystic Fibrosis Center Utrecht, University Medical Center Utrecht, Utrecht, The Netherlands

**Keywords:** Air pollution, Exposure assessment, Carotid artery, Children

## Abstract

**Background:**

Air pollution has been shown to promote cardiovascular disease in adults. Possible mechanisms include air pollution induced changes in arterial wall function and structure. Atherosclerotic vascular disease is a lifelong process and childhood exposure may play a critical role. We investigated whether air pollution is related to arterial wall changes in 5-year old children. To this aim, we developed an air pollution exposure methodology including time-weighted activity patterns improving upon epidemiological studies which assess exposure only at residential addresses.

**Methods:**

The study is part of an existing cohort study in which measurements of carotid artery intima-media thickness, carotid artery distensibility, elastic modulus, diastolic and systolic blood pressure have been obtained. Air pollution assessments were based on annual average concentration maps of Particulate Matter and Nitrogen Oxides at 5 m resolution derived from the European Study of Cohorts for Air Pollution Effects. We defined children’s likely primary activities and for each activity we calculated the mean air pollution exposure within the assumed area visited by the child. The exposure was then weighted by the time spent performing each activity to retrieve personal air pollution exposure for each child. Time spent in these activities was based upon a Dutch mobility survey. To assess the relation between the vascular status and air pollution exposure we applied linear regressions in order to adjust for potential confounders.

**Results:**

Carotid artery distensibility was consistently associated with the exposures among the 733 5-years olds. Regression analysis showed that for air pollution exposures carotid artery distensibility decreased per standard deviation. Specifically, for NO_2,_ carotid artery distensibility decreased by − 1.53 mPa^− 1^ (95% CI: -2.84, − 0.21), for NO_x_ by − 1.35 mPa^− 1^ (95% CI: -2.67, − 0.04), for PM_2.5_ by − 1.38 mPa^− 1^ (95% CI: -2.73, − 0.02), for PM_10_ by − 1.56 mPa^− 1^ (95% CI: -2.73, − 0.39), and for PM_2.5absorbance_ by − 1.63 (95% CI: -2.30, − 0.18). No associations were observed for the rest outcomes.

**Conclusions:**

The results of this study support the view that air pollution exposure may reduce arterial distensibility starting in young children. If the reduced distensibility persists, this may have clinical relevance later in life. The results of this study further stress the importance of reducing environmental pollutant exposures.

**Electronic supplementary material:**

The online version of this article (10.1186/s12940-019-0487-1) contains supplementary material, which is available to authorized users.

## Background

Air pollution may have been related to as many as 4.2 million premature deaths in 2016 globally, 44% of which are due to cardiovascular disease [1]. Oxidative stress and inflammation are suggested to be an important link between air pollution and cardiovascular risk due to atherosclerosis [2–4]. Compared to adults, children are more sensitive to air pollution because they breathe in more air per unit body-weight and consequently more air pollution [5]. The impact of air pollution is even more severe for children as their bodies are developing [6]. Additionally, children are exposed to higher concentrations of air pollution because their shorter stature results in them inhaling air from lower heights where some pollutants are in higher concentrations [6]. Thus, air pollution may impact cardiovascular health already early in life [7–10]. An emerging number of epidemiological studies have observed air pollution related changes in the carotid artery in adults [11–15] and young adults [16]. However, only one study has been reported in young children [17]. Carotid measurements suggest an end organ, non-invasive vascular detection of early signs of atherosclerosis in children. Moreover, there is evidence that there is a relation between tobacco smoke and carotid changes in the young [18]. As the mechanism which is hypothesized to lead to carotid changes from tobacco smoke is the same as with air pollution; we set out to determine the putative association between air pollutants and vascular characteristics in young children using a novel methodology that takes movements across various levels of exposure into account.

So far the methodology applied in large epidemiological studies, to estimate air pollution exposures, is mainly based on air pollution (point) estimates which either derived from individuals’ residential addresses [16, 19–22], or from schools’ addresses [23], or from the combination of the afore mentioned [8]. However, this might not be representative of the true exposure because humans are not static and do not spend their entire day at a fixed location [24]. Thus, person’s displacements are important to consider since air pollution differs considerably between areas where the activities take place [25–27]. Personal exposure can be described as the time-weighted average air pollution concentration of all activities of a person [28]. For this reason, there is a need to apply improved exposure assessment methods in epidemiological studies [29, 30] when sensor data are not available [31].

Our first aim was to model air pollution exposure based on a time-weighted activity pattern and detailed spatial maps of air pollution. Next, we assessed the relation between multiple air pollution exposures (PM_10_, PM_2.5_, PM_2.5absorbance_, NO_x_ and NO_2_) and cardiovascular markers in 5-yearold children.

## Subjects and methods

### Study population

**Fig. 1:**
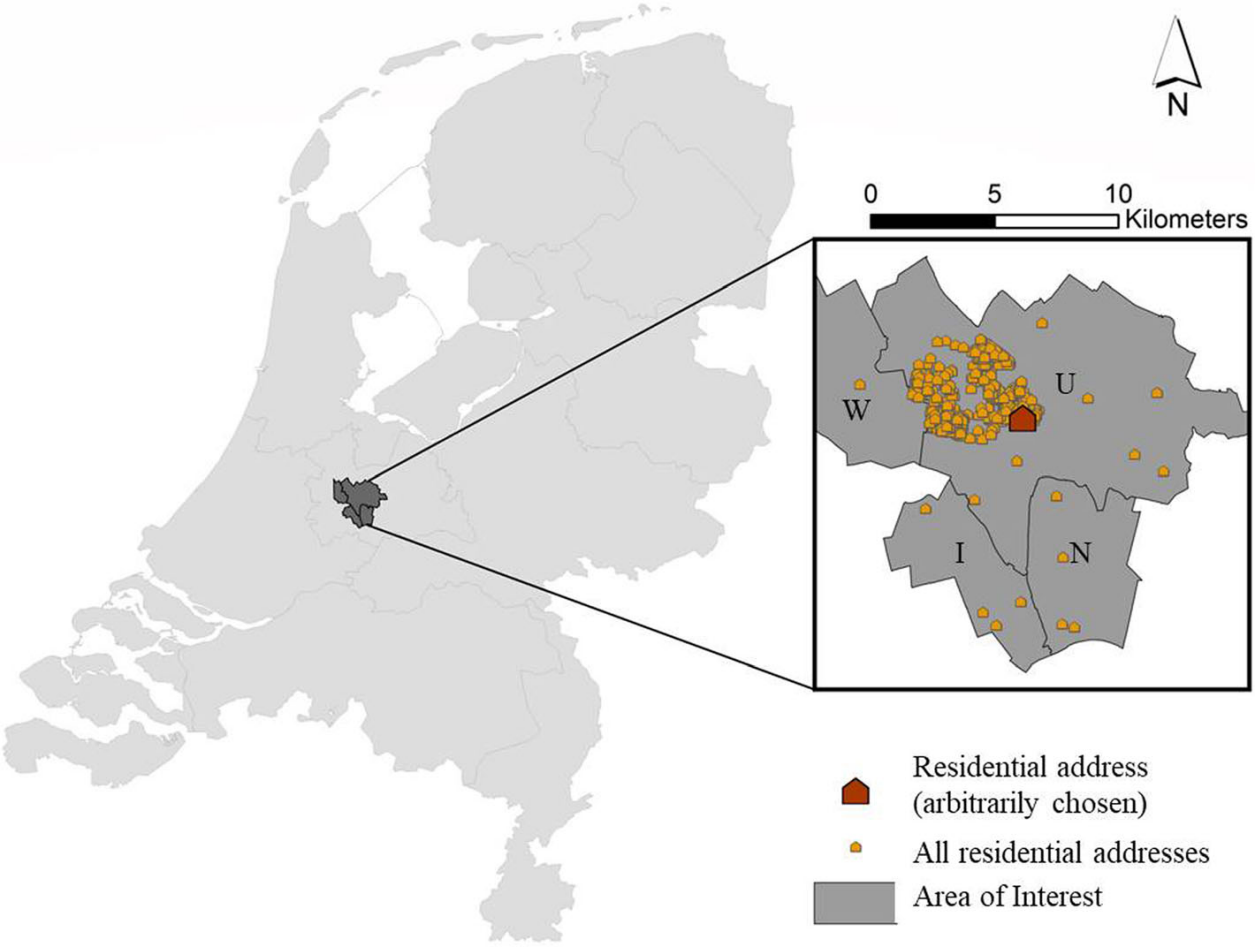
Area of interest and children’s residential addresses; mostly living in Leidsche Rijn, Utrecht Municipality (U), while some registries derive also from the neighboring municipalities (IJsselstein: I, Nieuwegein: N and Woerden: W)

This study is part of the Wheezing Illnesses Study Leidsche Rijn (WHISTLER), an ongoing population based prospective birth cohort study [32]. Virtually all participants are living in a 25 km^2^ residential area in the north-west of the Utrecht metropolitan area or nearby areas of Utrecht Municipality (Fig. 1).

The study was initiated in 2001 with a focus on lung disease and expanded in 2007 to include a range of measurements on cardiovascular development [18]. The current analyses used data obtained when the children had reached 5-years of age, which included measurements of carotid artery wall structure, and function (carotid Intima Media Thickness, carotid distensibility and Elastic Modulus). The carotid artery variables were measured by ultrasonography using high-resolution echo-tracking technology (Art.laboratory, Esaote, Italy) as described previously [18].

### Outcome and potential confounders

In this analysis the outcomes studied are the vascular conditions of the right common carotid artery which were measured ultrasonographically as described elsewhere [18, 32]. Carotid Intima Media Thickness (cIMT), carotid Distensibility (cD) and Elastic Modulus (EM) per individual were used to assess the elastic properties of the carotid artery, including the blood pressure measurements (systolic and diastolic).

As potential confounders we included maternal smoking in pregnancy [18, 33] and exposure to tobacco smoke [34, 35] which are suggested to affect vascular determinants in childhood and can be also associated with the exposure [36]. Socio-economic status (SES) was considered possible confounder because of its putative relation with determinant and outcome [37]. Although it is reported that normal carotid arterial wall is unaffected by age and sex until a certain age (10-years), it is uncertain if the uniform results are due to the low sensitivity of the imaging methods [38] and as such differences were observed in adults we included age and sex as potential confounders [39]. The general confounders (age, sex) and cigarette smoke exposure were extracted from the questionnaire filled during the 5-years old visit. The maternal smoking in pregnancy and SES questions were gathered by a questionnaire filled in by the mother during baseline examination [32].

### Modelling the individual air pollution exposures

To assess air pollution levels at the home addresses we used land use regression (LUR) models. The models were originally developed in the European Study of Cohorts for Air Pollution Effects (ESCAPE) project and described elsewhere [40, 41]. The models provide the annual average concentrations of several air pollutants at any location in the study area in the year 2010, including NO_2,_ NO_x_, PM_10_, PM_2.5_ and PM_2.5absorbance_ [42].

To extract information about the time spent at each activity we used the Onderzoek Verplaatsingen in Nederland 2010 (OViN: Study on mobility in the Netherlands) dataset [43]. This dataset includes information about the mobility of 1847 children aged 4–6 years living in the Netherlands. The parents who participated created a log over one week containing information about the place of origin and destination, the time when transport takes place, the used means of transport and the travel motives for each movement of their child [43].

To define where the children’s activities took place we used land use data: TOP10NL and Basisregistratie Adressen en Gebouwen (BAG), which is the Dutch cadastral information. Both datasets were available at the Dutch National Spatial Data Infrastructure (SDI): the Publieke Dienstverlening Op de Kaart (PDOK) [44]. Finally, school locations in the study area were recorded by University of Groningen Open Data [45].

The WHISTLER dataset included the residential address for each child defined by street name and house number. To link the address information to air pollution we transformed the addresses to coordinates by geocoding using Nominatim -a search engine for OpenStreetMap (OSM) [46]. The OSM point data were retrieved in WGS84 datum (EPSG 4326). We have re-projected the OSM point data to the local coordinate system: Amersfoort RD New datum (EPSG 28992) to correspond with the rest of the datasets.

To calculate each child’s individual exposures we defined their primary activities (being at home, playing in the neighbourhood, travelling to/from school or other destinations, and being at school) and then we calculated individualized exposures using the following formula:

1 $$ {E}_{ij}=\frac{T_h{C}_{h\left(i,j\right)}+{T}_p{C}_{p\left(i,j\right)}+{T}_s{C}_{s\left(i,j\right)}+{T}_t{C}_{t\left(i,j\right)}}{1440} $$

In Eq. 1, *E* is the personal exposure (μg/m^3^), *C*_*h*_ the air pollution concentration (μg/m^3^) representative for the being at home activity, and *T*_*h*_ the time (minutes) spent at an activity place, *C*_*p*_ the air pollution concentration for playing in the neighbourhood, *T*_*p*_ the time spent playing in the neighbourhood, *C*_*s*_ the air pollution concentration at school, *T*_*s*_ the time spent at school or at other educational activities, *C*_*t*_ the air pollution concentration at the road network, *T*_*t*_ the time spent travelling, *i* is the child id from 1 up to 733 and *j* the air pollutant 1 up to 6. All time units were measured in minutes;the denominator represents the total number of minutes in a day.

**Fig. 2:**
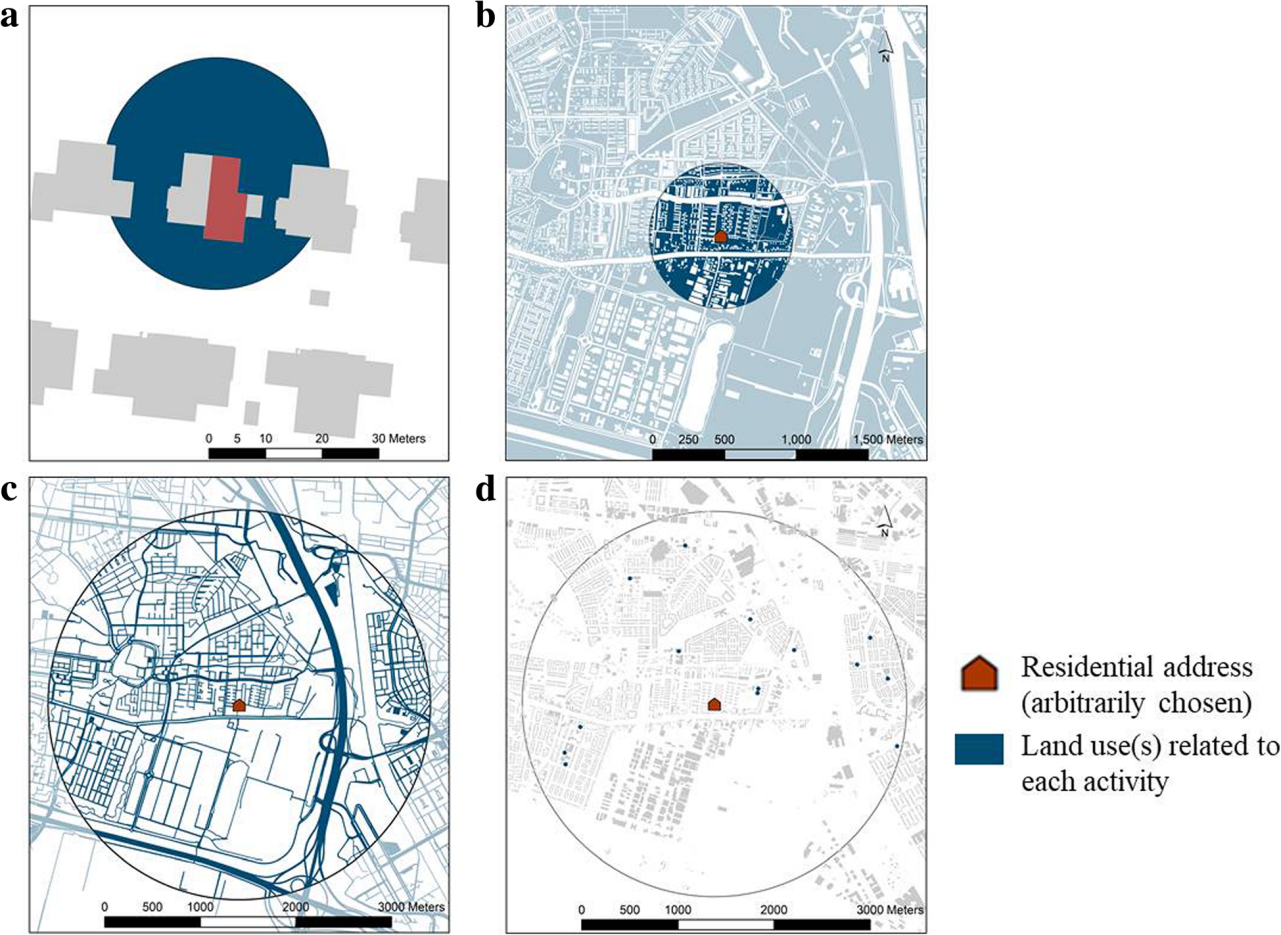
Representation of the buffers used, shown for a single (arbitrarily chosen) residential address (red) within the area of interest. Blue indicates the land use related to each activity (dark blue: intersecting polygons (within buffer), light blue: outside the buffer. **a** 20 m buffer around house to represent staying at home activity (note that the buffer is calculated around the location of the front door of the house, located at the upper-side in the figure), **b** 500 m buffer around house including open public and private space to represent the activity playing in the neighbourhood, **c** 2000 m buffer around the house including the road network to represent activities which include travelling, **d** 20 m buffer around schools which were included in the 2000 m buffer around the house to represent being at school activity

To compute the average air pollution concentration for each activity (*C*_*h*_, *C*_*p*_, *C*_*s*_, *C*_*t*_) we followed three primary steps. First, for each activity we defined the area where that activity can take place based on distance from home -by estimating the maximum distance away from home that a child would go during that particular activity. To define the buffer-sizes we made an educated guess based on the spatial scale of Leidsche Rijn. To represent the activity ‘being at home’ (*C*_*h*_) we used a 20 m buffer (Fig. 2a). To represent the activity ‘playing in the neighbourhood’ (*C*_*p*_), which includes activities such as playing at a nearby green area or visiting a neighbour, we used a 500 m buffer (Fig. 2b). A buffer of 2000 m was applied to represent activities which include travelling in the broader area, such as ‘travelling to/from school’ *(C*_*t*_*)* or following their parents to the super market (Fig. 2c). Finally, to represent the activity ‘being at school’ (*C*_*s*_) or to other educational activities, we applied 20 m buffers around all primary schools -by using the 20 m buffer we made sure that we included the complete educational building including their facilities-. Then, to identify the possible schools visited by each child we used a 2000 m buffer around each child’s house. For all schools within a 2000 m buffer we averaged their 20 m buffer air pollution concentrations (Fig. 2d).

Second, to estimate where a certain activity takes place, the land-uses were important to consider because there were certain land-use types that were not accessible (e.g. railways and industrial areas) or were not related to the activity we wanted to represent (e.g. cemeteries). Thus, we created maps of the area that could be visited during a particular activity as a function of land-use by producing maps that indicate the areas that can be reached as a function of distance from home location (step 1). For the activity staying at home (*C*_*h*_) we did not remove any land-uses (Fig. 2a). For the activity playing in the neighbourhood (*C*_*p*_) we computed the union of all possible land-uses from TOP10NL where a child can play (mainly open public and private spaces) (Fig. 2b). For the travelling activity (*C*_*t*_), the road network layer was used (Fig. 2c). For the activity being at school (*C*_*s*_) we used only the school areas (Fig. 2d).

Third, for each child and for each activity we created the intersection between the area that can be reached based on distance (step 1) and the area that will be visited during a particular activity (step 2). The intersection resulted in a map with the area visited during a particular activity for each child. This was done by first rasterizing (5 m cell size) the vector information generated in step 1 and 2 and executing their intersection.

Then, for each activity place and for each child, we calculated the average air pollution (respectively *C*_*h*_, *C*_*p*_, *C*_*s*_, *C*_*t*_) by averaging the air pollution concentration within the area that was visited during the activity (calculated in step 3), assuming that the presence of the child was  uniformly distributed over the area representing an activity. This was done by first calculating air pollution concentration levels over the entire study area -by applying the LUR models, derived from the ESCAPE models- and then averaging the values over the area which represented the activity for each child.

Finally, we used the OViN dataset to calculate the predicted average time children spent performing each of the primary activities (Eq. 1). To be able to deduct to the children participated in WHISTLER we selected all children between 4 and 6 year olds from the OViN dataset.. One of the assumptions we used in the model was the duration children spent at each activity place because this information was not available in the Whistler cohort. Therefore, we obtained this information from 4 to 6 years old children in the OViN study which is a random sample from the Dutch population and assumed that the children in the Whistler cohort would spend the same time at each activity place.

We found that children spent on average spent 964 min at home (*T*_*h*_) per day, in addition to this 28 min were spent playing in the neighbourhood (*T*_*p*_), 49 min travelling to school or travelling to other everyday activity (*T*_*t*_) and 399 min being at school and at other educational activities (*T*_*s*_).

All spatial computations based on vector files were performed using ArcGIS 10.4.1. and the raster calculations were performed in the PCRaster environment [47].

### Data analyses

We fitted models for all dependent variables (carotid Intima Media Thickness: cIMT, carotid distensibility: cD, Elastic Modulus: EM, Diastolic Blood Pressure: DBP and Systolic Blood Pressure: SBP) and for all personal exposures (NO_2,_ NO_x_, PM_10_, PM_2.5_ and PM_2.5absorbance_) calculated using Eq. 1. The exposures were entered as continuous variables. We tested the regression assumptions including linearity and found no deviation from linearity. We therefore used linear regression to obtain the association and the 95% confidence interval (CI) between the air pollutants and health variables.

We first fitted unadjusted models (Model 0) and then we adjusted for possible confounders by specifying four models with increasing levels of adjustment. In the first adjusted model (Model 1) we included sex and age. In the second model (Model 2a) we additionally adjusted for individual socio-economic status (SES) of parents (parental SES definition; 0: none of the parents was highly educated, 1: one of the parents was highly educated (university degree), 2: both of parents were highly educated). Finally, in Model 2b we added smoking of mother during pregnancy: Did you smoke during the pregnancy? (yes/no) and exposure to smoke in later life: Is your child exposed to smoke? (‘yes’, ‘no or not anymore’) in Model 2a. Finally, we performed subgroup analysis to the fullest model based on sex.

The confounding variables: sex, smoking of mother during pregnancy, smoking next to the child and individual parental SES were entered as categorical variables while age was entered as a continuous variable (Table 1). Observations with missing values for a variable were dropped from models including that variable. Specifically, we started with all the observations in Model 0 and Model 1 but approximately 16% of observations were lost in the most extensive confounder model (Model 2b) because values were missing for one or more of the confounders. The statistical analysis was performed using R version 3.5.0.

**Table 1:** Whistler cohort characteristics [*n* (%) of nonmissing observations], total population and stratified by sex

Characteristic	*n* (%) or mean (SD)			
Child characteristic	Total	Girls	Boys	Not available
Sex	733	376 (51.3)	357 (48.7)	0
Age (years)	5.42 (0.4)	5.41 (0.4)	5.42 (0.3)	0
Height (cm)	115.0 (4.8)	114.58 (4.8)	115.45 (4.7)	117
BMI	15.2 (1.4)	15.06 (1.4)	15.36 (1.4)	117
SBP (mmHg)	105.00 (7.5)	105.03 (7.5)	104.98 (7.5)	11
DBP (mmHg)	54.4 (7.2)	54.29 (7.4)	54.43 (7.1)	11
cIMT (μm)	385.6 (39.5)	381.8 (37.5)	389.3 (40.9)	73
EM (kPa)	159.3 (49.0)	156.2 (46.0)	162.6 (52.0)	187
cD (MPa^−1^)	81.0 (13.0)	80.1 (13.0)	81.97 (13.0)	157
Parental Characteristic				
Parental higher SES				178
None	132 (23.7)	64 (21.8)	68 (26.0)	
One parent	157 (28.3)	86 (29.4)	71 (27.1)	
Both parents	268 (48.0)	143 (48.8)	123 (46.9)	
Exposed to smoke during pregnancy	51 (7.0)	26 (7.0)	25 (7.1)	12
Exposed to smoke later in life	44 (6.1)	25 (6.8)	19 (5.4)	18
Exposures				
NO_2_ (μg/m^3^)	29.47 (2.1)	29.47 (2.1)	29.47 (2.1)	0
NO_x_ (μg/m^3^)	35.34 (6.2)	35.20 (6.1)	35.50 (6.3)	0
PM_2.5_ (μg/m^3^)	16.71 (0.2)	16.71 (0.2)	16.72 (0.2)	0
PM_10_ (μg/m^3^)	25.03 (0.6)	25.03 (0.6)	25.04 (0.6)	0
PM_2.5 absorbance_ (10^−5^/m)	1.32 (0.1)	1.32 (0.1)	1.32 (0.1)	0

## Results

Data on 733 healthy young children (mean age, 5.42 years) were used in the analyses. 51% of the participants were girls. 7.0% of children were exposed to cigarette smoke during pregnancy and 6.1% later in life (Table 1).

**Fig. 3:**
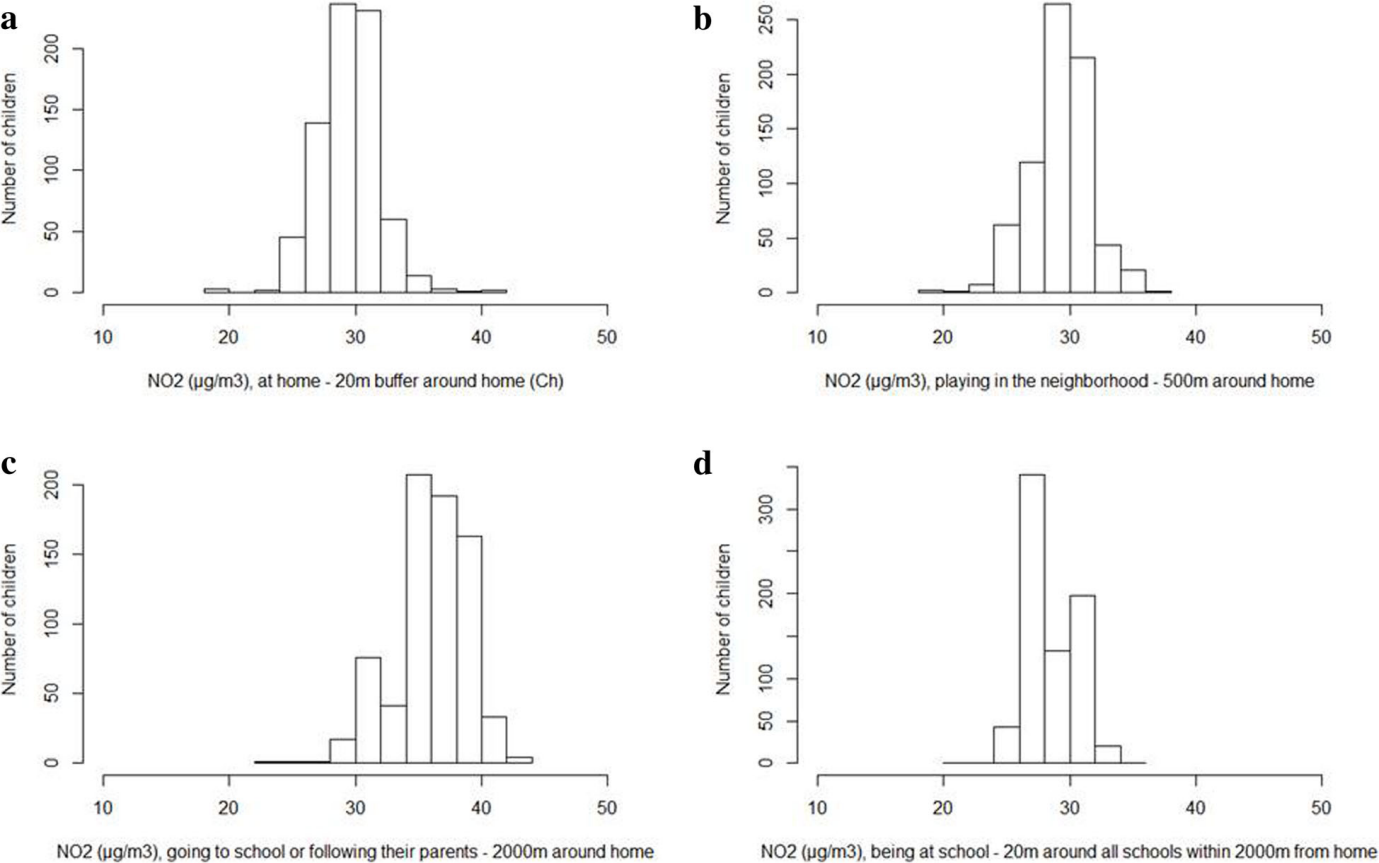
NO_2_ distributions during children’s primary activities. **a**, NO_2_ at home – 20 m buffer around home location (*C*_*h*_); **b**, NO_2_ playing in the neighbourhood – 500 m around home location (*C*_*p*_); **c**, NO_2_ going at school or following parents – 2000 m around home location (*C*_*t*_); **d**, NO_2_ being at school – 20 m around all schools within 2000 m around home (*C*_*s*_)

**Fig. 4:**
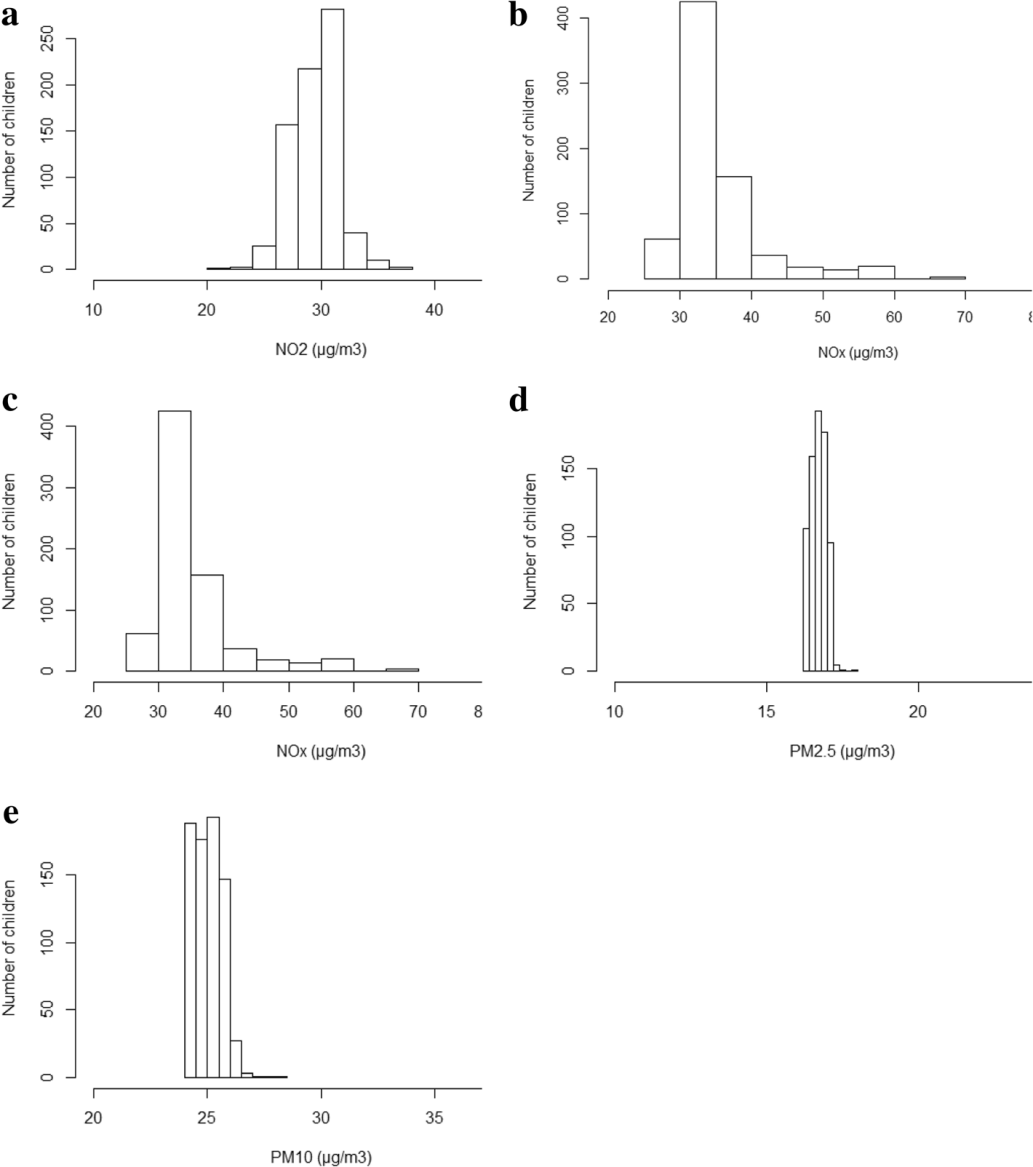
Distributions of air pollution exposures (*E*_*j*_), calculated from Eq. 1. **a** NO_2_ distribution; **b**.NO_x_ distribution; **c** PM_2.5_ distribution; **d** PM_10_ distribution; **e** PM_2.5absorbance_ distribution

Air pollution concentrations of the five air pollutants for each activity are presented in Table 3. Contrasts in exposures were moderate for NO_2_ and NO_x_ while for PM_2.5_ and PM_10_ contrasts were rather limited (Table 3). Concentration levels differ considerably between the activities for all air pollutants (Fig. 3). For all air pollutants the 2000 m buffer, which includes the road network to represent the activity ‘travelling to every-day activities’, had the highest concentration (Table 3); the distributions of the exposures are presented in Fig. 4. Correlations between the time-weighted activity pattern exposures (E) and front door exposure (C_d_) were above 0.84 with substantial scatter for NO_2_ (Additional file 1: Figure A1) as well as the correlations of the five air pollutants (Tables 2 and 3).

**Table 2:** Correlations coefficients of air pollution exposures

cor	NO_2_	NO_x_	PM_2.5_	PM_10_	PM_2.5abs_
NO_2_	1	0.49	0.46	0.79	0.80
NO_x_		1	0.60	0.70	0.70
PM_2.5_			1	0.69	0.66
PM_10_				1	0.98
PM_2.5abs_					1

**Table 3:** Air pollution concentrations for each activity/buffer

		Mean	SD	Median	Min	Max
NO_2_ (μg/m^3^)	*C*_*d*_	28.9	2.7	28.9	19.5	43.6
	*C*_*h*_	29.4	2.4	29.4	19.5	40.4
	*C*_*p*_	29.2	2.4	29.6	19.5	36.5
	*C*_*t*_	36.0	2.9	36.3	22.8	43.1
	*C*_*s*_	28.8	2.0	28.0	20.5	34.1
	*E*	29.4	2.1	29.6	20.1	37.9
NO_x_ (μg/m^3^)	*C*_*d*_	33.6	8.6	30.6	26.6	78.6
	*C*_*h*_	33.7	8.6	30.7	26.6	78.1
	*C*_*p*_	37.6	9.1	34.2	26.9	72.9
	*C*_*t*_	56.1	7.6	56.9	29.5	82.0
	*C*_*s*_	36.2	4.1	35.6	28.4	47.5
	*E*	35.2	6.1	33.9	27.8	69.6
PM_2.5_ (μg/m^3^)	*C*_*d*_	16.7	0.3	16.5	16.2	18.4
	*C*_*h*_	16.7	0.3	16.5	16.2	18.2
	*C*_*p*_	16.7	0.3	16.7	16.2	17.9
	*C*_*t*_	17.3	0.3	17.3	16.3	18.6
	*C*_*s*_	16.8	0.2	16.8	16.3	17.3
	*E*	16.7	0.2	16.7	16.2	17.9
PM_10_ (μg/m^3^)	*C*_*d*_	24.9	0.7	24.9	24.0	28.7
	*C*_*h*_	24.9	0.7	24.9	24.0	28.7
	*C*_*p*_	25.2	0.8	25.0	24.0	28.5
	*C*_*t*_	26.5	0.9	26.6	24.1	28.9
	*C*_*s*_	25.0	0.6	24.7	24.0	26.6
	*E*	25.0	0.6	25.0	24.1	28.1
PM_25 absorbance_ (10^−5^/m)	*C*_*d*_	1.3	0.1	1.3	1.2	2.0
	*C*_*h*_	1.3	0.1	1.3	1.2	1.9
	*C*_*p*_	1.3	0.1	1.3	1.2	1.8
	*C*_*t*_	1.6	0.1	1.6	1.2	2.1
	*C*_*s*_	1.3	0.1	1.3	1.2	1.6
	*E*	1.3	0.1	1.3	1.2	1.8

*C*_*d*_ is the air pollution level at front door location, *C*_*h*_ represents the air pollution concentration for the activity being at home, *C*_*p*_ the air pollution concentration for the activity playing in the neighbourhood, *C*_*t*_ for travelling and *C*_*s*_ for being at school, *E* represents the individual exposure after applying Eq. 1

We observed statistically significant associations between air pollutants and carotid artery distensibility (Table 4). In model 0 (unadjusted model) and model 1 (adjusted for age and sex) only PM_10_ and PM_2.5absorbance_ were associated with carotid artery distensibility (Table 4), whereas we observed an association for all air pollutants in model 2a and 2b. The regression slope of the full model (2b) was − 1.53 mPa-1 per SD NO_2_ increase (95% CI: -2.84, − 0.21), − 1.35 mPa-1 per SD NO_x_ increase (95% CI: -2.67, − 0.04), − 1.38 mPa^-1^ per SD PM_2.5_ increase (95% CI: -2.73, − 0.02), − 1.56 mPa-1 per SD PM_10_ increase (95% CI: -2.73,-0.39), and - 1.63 mPa^-1^ per SD PM_2.5absorbance_ increase (95% CI -2.30,-0.18). No statistically significant associations between the air pollutants and carotid artery intima-media thickness, elastic modulus and diastolic and systolic blood pressure were observed. Subgroup analysis for the fullest model stratified by sex showed stronger association for cD for boys but the difference in effect estimates were not statistically significant (Additional file 2: Table S1).

**Table 4:** Regression slopes (95% CI) for the associations (per SD = 1 increase) between air pollutant and children’s health depending on the level of confounder adjustment (in bold the significant associations, *p* < 0.05)

	Model0		Model1		Model2a		Model2b	
cIMT								
NO_2_	0.77	(−2.2, 3.8)	1.22	(−2.0, 4.4)	1.25	(−2.5, 5.0)	1.18	(−2.6, 5.0)
NO_x_	−0.42	(−3.4, 2.5)	− 0.25	(− 3.5, 3.0)	−1.47	(−5.3, 2.3)	−1.32	(−5.2, 2.5)
PM_2.5_	− 0.63	(− 3.6, 2.4)	−1.49	(−4.8, 1.8)	−1.87	(− 5.6, 1.9)	−1.57	(− 5.4, 2.3)
PM_10_	0.60	(−2.4, 3.6)	0.84	(−2.4, 4.1)	− 0.48	(−4.2, 3.2)	− 0.54	(− 4.3, 3.2)
PM_2.5abs_	0.42	(− 2.6, 3.4)	0.58	(−2.7, 3.8)	− 0.58	(−4.3, 3.1)	− 0.63	(− 4.4, 3.1)
EM								
NO_2_	−1.15	(−5.2, 2.9)	−2.62	(−7.1, 1.8)	− 1.01	(− 6.2, 4.2)	− 1.27	(− 6.5, 4.0)
NO_x_	− 1.92	(− 5.8, 2.0)	−2.29	(− 6.8, 2.2)	−2.43	(−7.5, 2.6)	− 2.14	(− 7.3, 3.0)
PM_2.5_	2.01	(−2.1, 6.1)	3.48	(− 1.1, 8.1)	4.26	(− 1.0, 9.5)	4.65	(− 0.7, 10)
PM_10_	− 1.45	(− 5.5, 2.6)	− 2.44	(−6.9–2.0)	−1.67	(− 6.7, 3.4)	− 1.91	(− 7.0, 3.2)
PM_2.5abs_	− 1.28	(− 5.3, 2.8)	−2.27	(−6.8, 2.2)	− 1.68	(− 6.8, 3.4)	−1.94	(− 7.1, 3.2)
cD								
NO_2_	−0.79	(−1.8, 0.2)	− 0.80	(− 1.8, 0.2)	**− 1.50**	**(− 2.8, − 0.2)**	**−1.53**	**(− 2.8, − 0.2)**
NO_x_	−0.90	(− 1.9, 0.1)	− 0.92	(−1.9, 0.1)	**− 1.33**	**(− 2.6, − 0.1)**	**−1.35**	**(− 2.7, − 0.1)**
PM_2.5_	−0.54	(− 1.6, 0.5)	−0.60	(− 1.7, 0.5)	**−1.34**	**(− 2.7, − 0.0)**	**−1.38**	**(− 2.7, − 0.0)**
PM_10_	**− 1.21**	**(− 2.3- -0.2)**	**−1.22**	**(− 2.3, − 0.2)**	**−1.44**	**(− 2.7, − 0.2)**	**−1.56**	**(−2.7, − 0.4)**
PM_2.5abs_	**− 1.25**	**(− 2.3- -0.2)**	**− 1.25**	**(− 2.3, − 0.2)**	**− 1.48**	**(− 2.8, − 0.2)**	**−1.63**	**(− 2.8, − 0.4)**
DBP								
NO_2_	0.13	(−0.4, 0.7)	0.23	(−0.3, 0.8)	0.38	(−0.3, 1.0)	0.39	(−0.3, 1.1)
NO_x_	0.18	(−0.3, 0.7)	0.19	(−0.4, 0.8)	0.01	(−0.7, 0.7)	−0.07	(− 0.8, 0.6)
PM_2.5_	0.50	(−0.1, 1.0)	0.54	(−0.0, 1.1)	0.59	(−0.1, 1.3)	0.54	(−0.1, 1.2)
PM_10_	0.24	(−0.3, 0.8)	0.30	(−0.3, 0.9)	0.32	(−0.3, 1.0)	0.32	(−0.3, 1.0)
PM_2.5abs_	0.20	(−0.3, 0.7)	0.32	(−0.3, 0.9)	0.30	(−0.3, 1.0)	0.31	(−0.3, 1.0)
SBP								
NO_2_	0.09	(−0.5, 0.6)	0.23	(−0.3, 0.8)	0.26	(−0.4, 0.9)	0.27	(−0.4, 0.9)
NO_x_	−0.24	(−0.7, 0.3)	0.11	(−0.7, 0.5)	− 0.16	(− 0.8, 0.5)	−0.07	(− 0.8, 0.6)
PM_2.5_	0.28	(−0.3, 0.8)	0.27	(−0.0, 1.1)	0.31	(−0.4, 1.0)	0.36	(−0.3, 1.0)
PM_10_	0.11	(−0.4, 0.6)	0.21	(−0.4, 0.8)	0.22	(−0.4, 0.9)	0.27	(−0.4, 0.9)
PM_2.5abs_	0.13	(−0.4, 0.7)	0.32	(−0.2, 0.9)	0.30	(−0.3, 1.0)	0.30	(−0.4, 1.0)

Model 0: completely unadjusted; Model 1: adjusted for sex, age; Model 2a: model 1+ parental SES characteristics; Model 2b: model 2a + exposed to smoke during pregnancy + child exposed to smoke later in life.

## Discussion

In the present study -using detailed vascular measurements and improved air pollution exposures in a large group of young children- all pollutants showed adverse relationships with carotid arterial distensibility independent from confounding variables. We did not observe any association between air pollution exposures and carotid artery intima-media thickness, elastic modulus or diastolic and systolic blood pressure at this young age.

To appreciate these findings, we have to address some aspects of the present study. First, a primary strength of this study is the use of a sophisticated exposure assessment based on validated geo-data to derive information for the possible activity pattern of children. Second, we enriched air pollution exposure data with time in order to derive a time-weighted activity pattern with the air pollution maps at a fine spatial scale. Third, we were able to assess several air pollutants including particulate matter (PM_10_, PM_2.5_, PM_2.5absorbance_) and nitrogen oxides (NO_x_, and NO_2_). Finally, this study is the first to evaluate the associations between air pollution and early atherosclerotic markers in the age of 5-years old children.

Some limitations should be addressed. A common challenge in similar studies is the individual exposure assessment, particularly because of the high spatio-temporal variation in air pollution. In this study, we present a method that takes into account the air pollution spatial variation by integrating air pollution over multiple activity zones corresponding to particular daily activities of children. In principle, this should lead to more realistic air pollution exposure values as the values represent the areas visited for a certain time. This is not the case when front-door pollution values are used to represent individual exposures. However, our method is prone to uncertainties in the estimation of the activity zones as well as the time spend on each activity. Specifically, we had no information on child-specific individual location of and time spent in activities, but we used the mobility survey to derive likely patterns. Given that few epidemiological studies have actual data on time activity, our approach represents a realistic method to incorporate time activity data. This approach can be further improved by incorporating temporal and seasonal variation in air pollution as well as indoor air pollution estimates if the interest lies in other sources of pollution. Moreover, the exposure assessment can be improved if more data related to the exact spatio-temporal locations visited by the children are available [48]. This would lead to an approach that relies on fewer assumptions. Furthermore, the air pollution exposures were calculated by using ESCAPE models for the year 2010 while the health measurements were collected between 2007 and 2013. Thus, the air pollution datasets cover partially the health measurement timeframe. However, as supported by previous research, an annual average of a single year is representative for a larger time-frame because the distribution of air pollution is stable for up to 8 years [49, 50], thus the models can be considered valid for the complete timeframe (2007–2013). Additionally, we did not access the particle composition which would be informative in terms of specific toxicants. We were unable to correct for possible confounders related to maternal cardio-metabolic conditions and nutritional status due to data unavailability. Although there were missing values within the dataset, the small differences in effect estimates between the different models argue against important selection bias derived from missing data. This study showed that increased air pollution is adversely related to carotid artery distensibility. Arterial distensibility is a measure of the arterial ability to expand and contract with cardiac pulsation and relaxation [51]. A decrease in arterial distensibility (increased artery wall stiffness) is generally observed with ageing, is accelerated by a number of cardiovascular risk factors such as smoking and blood pressure elevation and promotes the occurrence of symptomatic cardiovascular disease [52, 53]. Impairment of arterial wall function typically occurs in an early stage of the atherosclerotic process before structural wall changes (cIMT) become detectable [16, 54].

A previous study from Iannuzzi et al. (2010), which included 52 children aged 6 to 14 years, similarly reported a relation between air pollution exposure and carotid intima stiffness –in contrast no relation was found for arterial thickness. In addition, they observed no association between air pollution exposure and diastolic blood pressure. These results are all in line with the findings of this current study. In contrast, associations between air pollution exposure and higher blood pressure have been observed in a number of studies with children between 8 and 12 years [8, 23, 55]. From the stratified analysis based on sex analysis we did not observe differences between sexes.

We did not observe an association between air pollution exposure and structural arterial wall changes as measured by ultra-sonographic measurements of carotid intima media thickness. The most likely explanation is the young age of the participants, as structural abnormalities have not yet developed and therefore cannot be detected. No previous studies have attempted to relate air pollution to carotid artery intima-media thickness at such a young age. In adults though, there is evidence of structural arterial wall changes due to air pollution exposure [11, 56].

Another explanation is that the level of exposure was too low. Exposure levels were moderate compared to other European countries [57] and low in the global context [58]. Systematic inflammation is triggered by the increased levels of particulate matter and nitrogen oxides [3, 16]. Finally, there was low variation of especially PM_2.5_ and PM_10_ exposure in the study area. For NO_2_ and NO_x_ the contrast in exposure was moderate, consistent with previous work documenting that local sources affect NO_2_ more than PM_2.5_ [57]. Consistently, the confidence intervals for NO_2_ indicated that we were able to estimate air pollution effect sizes with good precision. The significant effect estimate for cD for NO_2_ translates into a 2–3% decrease per 1 SD.

The cross-sectional design does not allow detecting vascular changes through time as individual exposure to air pollution varied in different years. To elucidate more about the nature of the association and to be able to show causality a prospective study design is recommended. It would be of high interest to test the associations for children that have been exposed over a longer period to investigate if the associations persist, for example using the same population when they reach the adolescence stage. Furthermore, our exposure assessment method could be replicated in an area with greater air-pollution variation.

## Conclusions

The results of this study suggest that air pollution may contribute to vascular disease starting at a very young age. Therefore, it is likely that that early-life air pollution exposures might be the key to more effective strategies for prevention of cardio-vascular disease. In view of the enormous numbers of children facing lifelong exposure to environmental air pollution and the epidemic of cardiovascular disease, the findings stress the need for reductions in air pollution and reduction of individual exposure.

## Additional files

Additional file 1: **Figure S1.** Scatterplots showing the relationships between air pollution at the front door location (C_d_) and exposures calculated using the time-weighted activity pattern (*E*_*j*_), calculated from Eq. 1. a: the relationship between NO_2_ at front door location (C_d NO2_) and NO_2_ after applying Eq.1 (E_NO2_); b: the relationship between NO_x_ at front door location (C_d NOx_) and NO_x_ after applying Eq.1 (E_NOx_); c: the relationship between PM_2.5_ at front door location (C_d PM2.5_) and PM_2.5_ after applying Eq.1 (E_PM2.5_); d: the relationship between PM_10_ at front door location (C_d PM10_) and PM_10_ after applying Eq.1. (E_PM10_); e: the relationship between PM_2.5absorbance_ at front door location (C_d PM2.5absorbance_) and PM_2.5absorbance_ after applying Eq.1. (E_PM2.5absorbance_). (PDF 298 kb)

Additional file 2: **Table S1.** Regression slopes (95% CI) between air pollutants and cD for the fullest model (Model2b) stratified by sex. (DOCX 13 kb)

## Abbreviations

cD: carotid Distensibility
CI: Confidence Interval
cIMT: carotid Intima Media Thickness
DBP: Diastolic Blood Pressure
EM: Elastic Modulus
ESCAPE: European Study of Cohorts for Air Pollution Effects
LUR: Land Use Regression
NO_2_: Nitrogen Dioxide
NO_x_: Nitrogen Oxides
OViN: Onderzoek Verplaatsingen in Nederland (eng: Study on mobility in the Netherlands)
PM_10_: Particulate matters with diameter < 10 μm
PM_2.5_: Particulate matters with diameter < 2.5 μm
SBP: Systolic Blood Pressure
SD: Standard Deviation
SES: Socio-economic status
WHISTLER: Wheezing Illnesses Study Leidsche Rijn
