# Supplementary figures and images for: Relations between air pollution and vascular development in 5-year old children: a cross-sectional study in the Netherlands
Source: Environ Health. 2019 May 16;18:50. doi: 10.1186/s12940-019-0487-1 (PMC6524285; doi:10.1186/s12940-019-0487-1)

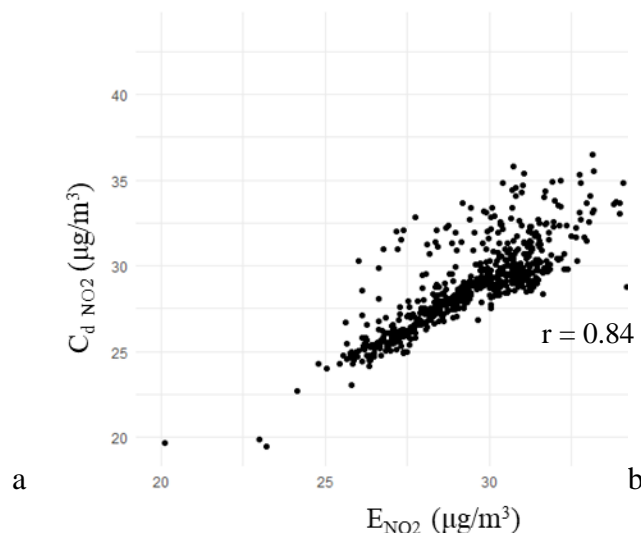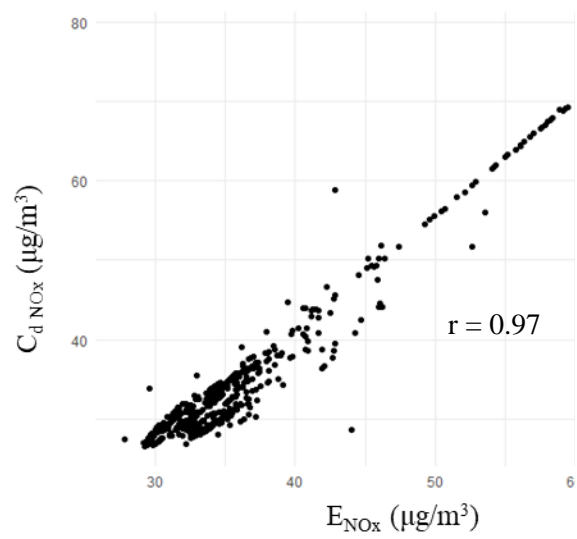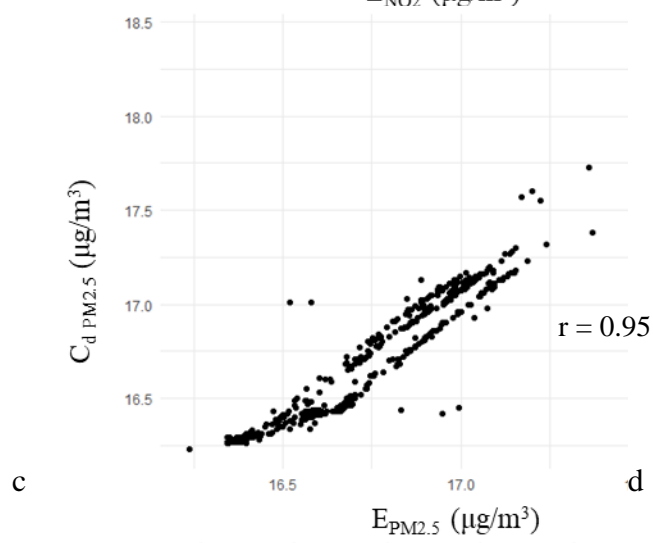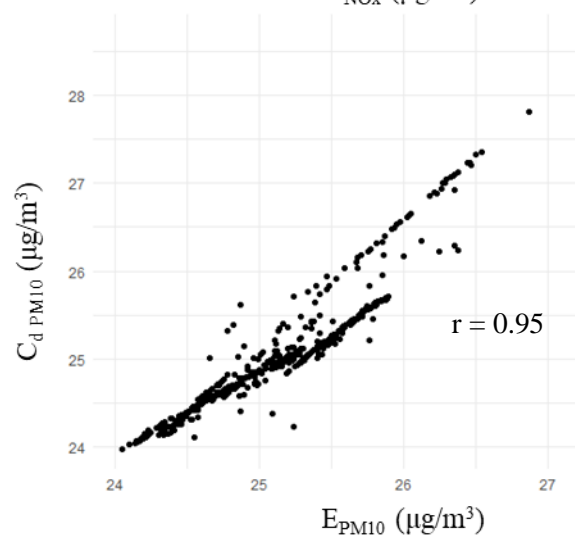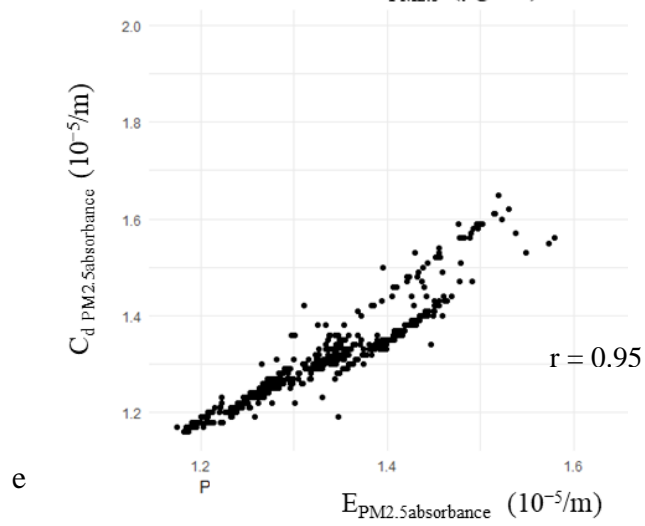

Supplement: Supplementary file 1 — Figure S1. Scatterplots showing the relationships between air pollution at the front door location (Cd) and exposures calculated using the time-weighted activity pattern (Ej), calculated from Eq. 1. a: the relationship between NO2 at front door location (Cd NO2) and NO2 after applying Eq.1 (ENO2); b: the relationship between NOx at front door location (Cd NOx) and NOx after applying Eq.1 (ENOx); c: the relationship between PM2.5 at front door location (Cd PM2.5) and PM2.5 after applying Eq.1 (EPM2.5); d: the relationship between PM10 at front door location (Cd PM10) and PM10 after applying Eq.1. (EPM10); e: the relationship between PM2.5absorbance at front door location (Cd PM2.5absorbance) and PM2.5absorbance after applying Eq.1. (EPM2.5absorbance). (PDF 298 kb) [file 12940_2019_487_MOESM1_ESM.pdf]
