# Supplementary material for: Relations between air pollution and vascular development in 5-year old children: a cross-sectional study in the Netherlands
Source: Environ Health. 2019 May 16;18:50. doi: 10.1186/s12940-019-0487-1 (PMC6524285; doi:10.1186/s12940-019-0487-1)
Supplement: Supplementary file 2 — Table S1. Regression slopes (95% CI) between air pollutants and cD for the fullest model (Model2b) stratified by sex. (DOCX 13 kb) [file 12940_2019_487_MOESM2_ESM.docx]

| **cD** | **Model2b Boys** | | **Model2b Girls** | |
| --- | --- | --- | --- | --- |
| NO_2_ | -1.84 | (-3.9, 0.2) | -1.3 | (-3.0, 0.4) |
| NO_x_ | -1.65 | (-3.5, 0.2) | -1.11 | (-3.0, 0.8) |
| PM_2.5_ | -1.89 | (-3.9, 0.2) | -0.90 | (-2.7, 0.9) |
| PM_10_ | **-1.94** | **(-3.8, -0.0)** | -1.00 | (-2.7, 0.7) |
| PM_2.5abs_ | **-2.12** | **(-4.0, -0.2)** | -0.93 | (-2.7, 0.8) |
